# Amiloride‐sensitive fluid resorption in NCI‐H441 lung epithelia depends on an apical Cl^−^ conductance

**DOI:** 10.1002/phy2.201

**Published:** 2014-01-16

**Authors:** Jonas P. Korbmacher, Christiane Michel, Daniel Neubauer, Kristin Thompson, Boris Mizaikoff, Manfred Frick, Paul Dietl, Oliver H. Wittekindt

**Affiliations:** 1 Institute of General Physiology, Ulm University, Albert‐Einstein‐Allee 11, Ulm, 89081, Germany; 2 Institute of Analytical and Bioanalytical Chemistry, Ulm University, Albert‐Einstein‐Allee 11, Ulm, 89081, Germany

**Keywords:** Cl^−^ channels, epithelial transport, fluid resorption, lung

## Abstract

Proper apical airway surface hydration is essential to maintain lung function. This hydration depends on well‐balanced water resorption and secretion. The mechanisms involved in resorption are still a matter of debate, especially as the measurement of transepithelial water transport remains challenging. In this study, we combined classical short circuit current (*I*_SC_) measurements with a novel D_2_O dilution method to correlate ion and water transport in order to reveal basic transport mechanisms in lung epithelia. D_2_O dilution method enabled precise analysis of water resorption with an unprecedented resolution. NCI‐H441 cells cultured at an air–liquid interface resorbed water at a rate of 1.5 ± 0.4 *μ*L/(h cm^2^). Water resorption and *I*_SC_ were reduced by almost 80% in the presence of the bulk Cl^−^ channel inhibitor 5‐nitro‐2‐(3‐phenylpropylamino)benzoic acid (NPPB) or amiloride, a specific inhibitor of epithelial sodium channel (ENaC). However, water resorption and *I*_SC_ were only moderately affected by forskolin or cystic fibrosis transmembrane regulator (CFTR) channel inhibitors (CFTR_inh_‐172 and glybenclamide). In line with previous studies, we demonstrate that water resorption depends on ENaC, and CFTR channels have only a minor but probably modulating effect on water resorption. However, the major ENaC‐mediated water resorption depends on an apical non‐CFTR Cl^−^ conductance.

## Abstract (precis)

We investigated water transport across lung epithelia, using a novel D_2_O dilution method in combination with Ussing chamber experiments. Our results revealed that CFTR channels have a minor modulating effect on water resorption. The major ENaC‐mediated resorption depends on non‐CFTR channels.

## Introduction

Transepithelial water transport occurs by osmosis, following mainly the net transport of NaCl. The direction of net NaCl transport (resorption vs. secretion) results from targeted expression of Na^+^/K^+^‐ATPases and ion channels and transporters in polarized epithelia. The epithelial sodium channel (ENaC) and the cystic fibrosis transmembrane regulator (CFTR) are the most intensively investigated ion channels and transporters in pulmonary epithelia.

Na^+^ uptake across the apical membrane is the rate‐limiting step in fluid resorption of lung, which is predominantly mediated by apically localized ENaC (Hummler et al. 1996; Elias et al. 2007). Apically localized Cl^−^ conductance in turn is postulated to drive fluid secretion in fetal (O'Brodovich 2001) and mature lung (Lindert et al. 2007). The paradigm that CFTR activity is associated with Cl^−^ and water secretion does not apply to all epithelial cell layers. In duct cells of submandibular salivary glands, both CFTR and ENaC channels are essential for NaCl resorption (Catalán et al. 2010). Also, in lung epithelia Na^+^ resorption involves CFTR channels (Jiang et al. 1998). This early observation is in line with other studies, demonstrating that *β*‐adrenergic stimulation results in increased fluid resorption across distal lung epithelia via adenosin 3’,5’‐cyclic monophsophate (cAMP)‐mediated activation of CFTR (Fang et al. 2002; Mutlu et al. 2005; Eisenhut 2007). This frequently underestimated function of CFTR on resorptive transport pathways may be explained by transcellular Cl^−^ resorption, as well as by its hyperpolarizing effect on the apical membrane, which would facilitate Na^+^ uptake (O'Grady et al. 2000). It appears from these studies that apically localized Cl^−^ conductance per se can support resorptive transport processes. However, such a function was not yet considered for non‐CFTR Cl^−^ channels.

NCI‐H441 cells were recently introduced as a model for the alveolar epithelium (Hermanns et al. 2004). Although this is by definition a simplification, as the alveolar epithelium consists of two epithelial cell types, alveolar cell type I and II cells, NCI‐H441 cells share characteristics with native alveolar cells, especially when cultivated at an air–liquid interface (ALI): (1) these cells are expressing ENaC (Itani et al. 2002; Neubauer et al. 2013) and Cl^−^ channels (Kulaksiz et al. 2002), (2) formation of a polarized epithelium (Hermanns et al. 2004), and (3) active fluid resorption (Neubauer et al. 2013). In these epithelia the role of Cl^−^ channels is still a matter of debate. Cell models of resorptive epithelia generally relate the paracellular shunt pathway in conjunction with the lumen‐negative transepithelial electrical potential difference with Cl^−^ resorption (Kim et al. 1991). However, the role of apically localized Cl^−^ channels is still unclear.

We have recently established a novel D_2_O dilution method to investigate transepithelial water transport, which enables correlating water and ion transport across epithelial cell layers (Neubauer et al. 2013). Using this method, we have now studied the impact of apically localized Cl^−^ conductance on water resorption in NCI‐H441 cell layers demonstrating that ENaC provides the major driving force for transepithelial water transport. Moreover, we show that ENaC‐mediated transport is not only modulated by apical Cl^−^ channel activity, but actually depends on it.

## Materials and Methods

### Cell culture

NCI‐H441 cells were obtained from American Type Culture Collection (ATCC, Manassas, VA) and cultivated in 25 cm² flasks together with 10 mL of culture medium (RPMI 1640 medium; Pan Biotech, Aidenach, Germany) containing 2.5 *μ*g/mL Na‐pyruvate (Sigma–Aldrich, Taufkirchen, Germany) and 10% charcoal stripped fetal calf serum (Sigma–Aldrich). On day 7, the cell layers reached 80% confluence. For further measurements, the cells were suspended using Trypsin LE (Invitrogen, Darmstadt, Germany) according to the manufacturer's protocol; thereafter, 10^4^ cells were seeded onto 0.33 cm^2^ permeable polyester transwell filter inserts (Costar 3470; Corning, Fisher Scientific, Schwerte, Germany) and were placed in 24‐well plates. The apical compartment was filled with 200 *μ*L and the lower compartment with 500 *μ*L of culture medium. On day 4 after seeding, an ALI was established by removing the medium from the upper compartment. The medium of the lower compartment was replaced by ALI medium (culture medium: +30 nmol/L dexamethasone, 1.72 *μ*mol/L insulin, 68.8 *μ*mol/L transferrin, and 38.7 nmol/L sodium selenite [ITS; Invitrogen]). Medium was exchanged every second day. On days 6–7, the cells were able to maintain a stable ALI. Water transport and *I*_sc_ measurements were performed on day 11 after seeding.

### Water transport measurements

Water transport was measured using the D_2_O dilution method, a newly established method enabling a precise quantification of apical surface liquid volume on epithelial cell layers. Measurements were performed as previously described (Neubauer et al. 2013). In brief, filters with confluent cell layers were placed into the wells of a 24‐well plate. The basolateral compartment was filled with 500 *μ*L of ALI medium, and 25 *μ*L of isotonic NaCl solution was added to the apical compartment. To avoid evaporation, empty wells as well as the space in between the wells were filled with isotonic NaCl solution. To estimate volume changes caused by evaporation, silicon sealed control filters were loaded with 25 *μ*L isotonic NaCl solution and placed randomly on the same plate. Compounds were added to the basolateral ALI medium as well as to the isotonic NaCl solution that was added to the apical compartment at the mentioned concentrations. The cells were incubated for 16 h at 37°C, 5% CO_2_, and 95% humidity. The remaining apical volume was dissolved in 25 *μ*L D_2_O containing 0.9% (w/v) NaCl, and water concentrations were determined via attenuated total reflection midinfrared spectroscopy using a Vertex 70 Fourier transform infrared (FT‐IR) spectrometer equipped with a BioATR assembly and a liquid nitrogen–cooled mercury–cadmium–telluride detector (all IR devices Bruker Optics, Etlingen, Germany), as previously described (Neubauer et al. 2013). Water concentration was calibrated for 15%, 25%, 40%, 50%, and 65% (v/v) of water in D_2_O. Area below absorption bands (wave lengths as wave numbers per cm, H–O stretching band: 3810.6 cm^−1^–2805 cm^−1^; D–O stretching band: 2774 cm^−1^–2070cm^−1^; D–O–D bending band: 1818 cm^−1^–1090 cm^−1^) were blotted against water concentration and linear regression was calculated through data points to obtain the slope (*m*) and *y*‐interception (*y*_0_). Sample water concentrations were calculated according to: 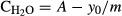 with 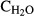 as water concentration of sample as %, *A* area under absorption band. The apical volume (*V*_api_) was calculated according to: 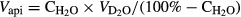 with 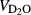 as volume of D_2_O with 0.9% NaCl in which the apical volume was diluted (in our case 25 *μ*L). Changes in apical volume (Δ*V*_api_) as well as for evaporation (Δ*V*_evap_) controls were calculated according to: Δ*V*_api_ = *V*_*t* = 0_ − *V*_api_ and Δ*V*_evap_ = *V*_*t* = 0_ − *V*_evap_ with *V*_api_ and *V*_evap_ as remaining apical volume and volume of evaporation control, respectively, and *V*_*t* = 0_ as volumes added to the apical side or to the evaporation control filter (in our case 25‐*μ*L isotonic NaCl solution) at time point 0. Apical volume change was corrected for evaporation by subtracting Δ*V*_cor_ = Δ*V*_api_− Δ*V*_evap_ with Δ*V*_cor_ as evaporation corrected apical volume. Transport rate (*F*) was calculated according to *F* = Δ*V*_cor_/(*t*_inc_·*M*), with *t*_inc_ = incubation time (in our case 16 h) and *M* = epithelial surface area (in our case 0.33 cm^2^). In order to account for variations between cell passages, data are given as relative water resorption calculated according to *F*_rel_ = *F*_sample_/*F*_control_, with *F*_sample_ as the water transport rate obtained from treated cells, and *F*_control_ as the averaged water transport rate determined for untreated control cells of matched cell passages.

### Ussing chamber experiments

Filters were inserted into easy‐mount diffusion chambers (Physiologic Instruments, San Diego, CA), and the temperature was adjusted to 37°C. A custom‐made amplifier was used for measurements. LabVIEW software package (National Instruments, München, Germany) was used for controlling and data acquisition. Apical and basolateral compartments were filled with 5 ml bath solution (BS, in mmol/L: 140 NaCl, 5 KCl, 10 HEPES, 1 KH_2_PO_4_, 1 MgSO_4_, 2.5 CaCl_2_, 10 glucose, pH 7.4) preheated to 37°C. Solutions were circulated by air gassing. To establish Cl^−^ gradients, Cl^−^ was replaced by gluconate either in the apical or basolateral compartment. Compounds were added at given concentrations to the respective compartment. The transepithelial potential was clamped to 0 mV and the short circuit current (*I*_sc_) was measured. For analyzing transepithelial electrical resistance, symmetrical voltage pulses of ±5 mV were applied every 10 sec. Relative *I*_sc_ was calculated according to ^rel^*I*_sc_ = *I*_sc_/^con^*I*_sc_ with *I*_sc_ as *I*_sc_ measured in the presence of compound and ^con^*I*_sc_ as *I*_sc_ measured before compounds were added.

### Statistical analysis

GraphPad Prism version 6.00 (GraphPad Software, La Jolla, CA) was used for statistical analysis. ANOVA test was performed followed by multiple comparisons using Holms–Sidak correction. Student's *t*‐test was performed for single comparison. Significant levels were indicated as follows: *P < 0.05; **P < 0.01; ***P < 0.001; ****P < 0.0001.

## Results

### Baseline water resorption is sensitive to amiloride

**Figure 1.**
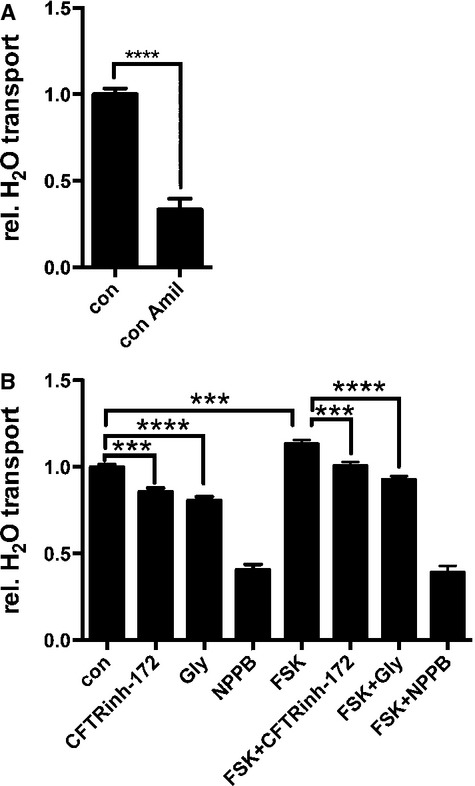
Pharmacological properties of water resorption in NCI‐H441 epithelia. (A) Amiloride inhibits water resorption. (B) Effect of Cl‐channel modulators on water resorption. Cells were cultivated in the presence of 30 nmol/L dexamethasone at air–liquid interface and experiments were performed as described. Con = control cells; amil = 30 *μ*mol/L amiloride; CFTR_inh_‐172 = 50 *μ*mol/L CFTR_inh_‐172; Gly = 100 *μ*mol/L glybenclamide; FSK = 100 *μ*mol/L forskolin. Compounds were added at given concentrations to basolateral and apical compartments.

Water transport across H441 monolayers was determined using the recently described D_2_O dilution method (Neubauer et al. 2013). This method enables the quantification of volume changes in extracellular aqueous solutions with an unprecedented precision. Under control conditions, H441 monolayers resorb water with an average transport rate of 1.5 ± 0.4 *μ*L/(h cm^2^). In order to account for variability in water transport between different cell batches, transport rates were normalized to the average transport rate under control conditions in the corresponding batch. According to generally accepted models of transepithelial transport, the rate‐limiting step for resorption is Na^+^ entry via the amiloride‐sensitive ENaC. Therefore, the effect of amiloride on water transport was investigated. At symmetrical Cl^−^ and Na^+^ concentrations, amiloride significantly reduces the relative water resorption by more than 70% to 0.33 ± 0.06 (mean ± SEM, *N* = 13, unpaired Student's *t*‐test *P* < 0.0001) (Fig. 1A). This confirms the rate‐limiting role of ENaC.

### Role of Cl^−^ channels for water resorption

Presently, the role of Cl^−^ channels for water resorption in lung epithelial is still unclear. Therefore, we investigated the effects of Cl^−^ channel modulators on water transport (Fig. 1B). The CFTR‐specific inhibitors CFTR_inh_‐172 and glybenclamide partially inhibited water resorption (0.86 ± 0.02, *P* < 0.001, and 0.81 ± 0.02, *P* < 0.0001, relative resorption in the presence of CFTR_inh_‐172 and glybenclamide, respectively, mean ± SEM, *N* = 15, ANOVA control vs. CFTR_inh_‐172 and glybenclamide). In contrast, 5‐nitro‐2‐(3‐phenylpropylamino)benzoic acid (NPPB), which blocks a wide range of Cl^−^ channels, reduced the relative water resorption by almost 60% to 0.41 ± 0.03 (mean ± SEM, *N* = 15). Thus, bulk blockage of Cl^−^ channels reduces water resorption with a similar efficacy of ENaC blockage via amiloride.

Forskolin (FSK) treatment to elevate intracellular cAMP significantly increased the relative water resorption to 1.13 ± 0.02 (mean ± SEM, *N* = 16, ANOVA FSK vs. control *P* < 0.001) (Fig. 1B). To test whether the FSK‐induced increase was due to CFTR activation, FSK stimulation in the presence of glybenclamide and CFTR_inh_‐172 was performed. Both CFTR inhibitors reduced relative water resorption in FSK‐stimulated epithelia to basal levels of untreated control cells (relative water resorption 1.007 ± 0.02 and 0.93 ± 0.04 for FSK + CFTR_inh_‐172 and FSK + Gly, respectively). In the presence of FSK, NPPB reduced the water resorption to 0.39 ± 0.02 (mean ± SEM, *N* = 16).

As expected, water resorption is driven and limited by amiloride‐sensitive ENaC‐mediated ion transport. Interestingly, NPPB‐sensitive Cl^−^ channels seem to have an equally rate‐limiting effect on water resorption. Furthermore, the experiments in this study demonstrate that water resorption may be modulated via cAMP‐dependent regulation of Cl^−^ channels, that is, most likely via CFTR. This observation agrees with previous studies (O'Grady et al. 2000; Mutlu et al. 2004, 2005; Fang et al. 2006).

**Figure 2.**
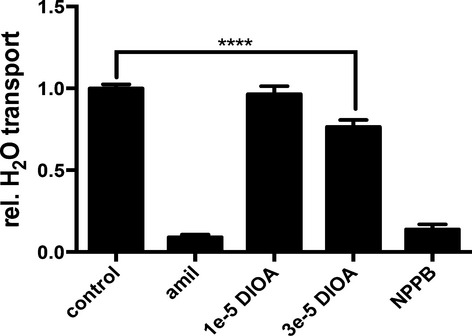
Effect of DIOA on water resorption. Cells were cultivated in the presence of 30 nmol/L dexamethasone at air–liquid interface and experiments were performed as described. Con = control cells; amil = 30 *μ*mol/L amiloride; DIOA = R‐(+)‐butylindazone (numbers represent concentrations in mol/L); NPPB = 200 *μ*mol/L 5‐nitro‐2‐(3‐phenylpropylamino)benzoic acid. Compounds were added at given concentrations to basolateral and apical sides.

In lung epithelia, transcellular Cl^−^ transport has been shown to depend on K^+^/Cl^−^ cotransporters of the SLC12A subfamily (Lee et al. 2003). Therefore, the effect of DIOA (R‐(+)‐Butylindazone) as a blocker of K^+^/Cl^−^ cotransporter on water resorption was tested. DIOA reduced the relative water resorption to 0.76 ± 0.04 (mean ± SEM, *N* = 18) at a concentration of 30 *μ*mol/L (Fig. 2). Concentrations above 30 *μ*mol/L were not tested, as they also block ATPases (Fujii et al. 2007).

### Effect of amiloride and NPPB on *I*_sc_

**Figure 3.**
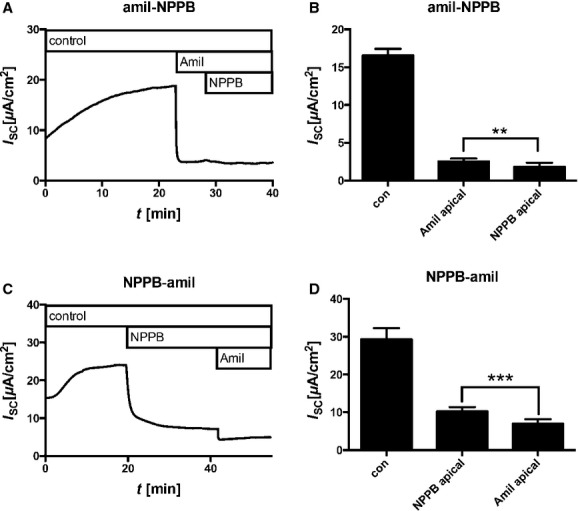
Effect of amiloride and NPPB on short circuit current (*I*_sc_). Cells were cultivated in the presence of 30 nmol/L dexamethasone at air–liquid interface, mounted into Ussing chambers, and *I*_SC_ measurements were performed. (A) Time course of representative *I*_SC_ measurement. (B) Bar diagram summarizes measured *I*_SC_ as mean ± SEM. NPPB showed hardly any effect when added in the presence of amiloride. (C) Time course of representative *I*_SC_ measurement. (D) Bar diagram summarizes measured *I*_SC_ as mean ± SEM. Inhibition of *I*_SC_ by amiloride was diminished in the presence of NPPB. Con = bath solution without any compound. Amil = 30 *μ*mol/L amiloride, NPPB = 200 *μ*mol/L 5‐ nitro‐2‐(3‐phenylpropylamino)benzoic acid. In all experiments, compounds were added solely to the apical compartment.

Ussing chamber experiments (Fig. 3A) revealed that amiloride (30 *μ*mol/L) caused a significant and substantial *I*_sc_ reduction (*I*_sc_ control 16.56 ± 0.9 *μ*A/cm^2^, amiloride 2.53 ± 0.4 *μ*A/cm^2^, mean ± SEM, *N* = 6, paired Student's *t*‐test *P* < 0.0001). Thus, *I*_sc_ can be mainly attributed to ENaC‐mediated ion transport. Subsequent addition of NPPB only moderately but significantly reduced *I*_sc_ (*I*_sc_ amiloride + NPPB 1.83 ± 0.5 *μ*A/cm^2^, mean ± SEM, *N* = 6, *t*‐test amiloride vs. amiloride + NPPB *P* = 0.0096). Similar to amiloride, the exclusive application of NPPB equally led to a major decrease in *I*_sc_ (Fig. 3B, *I*_sc_ control 29.28 ± 3.0 *μ*A/cm^2^, NPPB 10.24 ± 1.1 *μ*A/cm^2^, mean ± SEM, *N* = 7, paired Student's *t*‐test *P* < 0.0001). The subsequent addition of amiloride resulted in a further, yet minor *I*_sc_ reduction (*I*_sc_ NPPB + amiloride 7.00 ± 1.2 *μ*A/cm^2^, *N* = 7, paired Student's *t*‐test, NPPB vs. NPPB + amiloride *P* < 0.0001). These results are in line with the results from the water transport measurements.

### Effect of FSK on *I*_sc_

**Figure 4.**
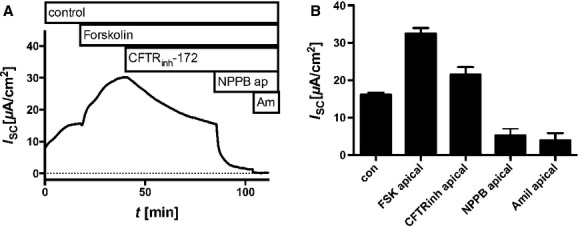
Effect of Cl^−^ channel modulators on short circuit currents (*I*_SC_). Cells were cultivated in the presence of 30 nmol/L dexamethasone at air–liquid interface, mounted into Ussing chambers, and *I*_SC_ measurements were performed. (A) Representative time course of *I*_SC_. Time intervals at which compounds were present are assigned as bars above. (B) Bar diagram summarizes measured *I*_SC_ as mean ± SEM. Compounds were added solely to apical side.

FSK (50 *μ*mol/L) induced a significant increase in *I*_sc_ (*I*_sc_ control 16.14 ± 0.57 *μ*A/cm^2^, FSK 32.53 ± 2.0 *μ*A/cm^2^ mean ± SEM, *N* = 6, paired Student's *t*‐test *P* < 0.0001 control vs. FSK) (Fig. 4). The FSK‐activated *I*_sc_ was sensitive to CFTR_inh_‐172. Evidently, the FSK‐induced water resorption and increase in *I*_sc_ are dependent on CFTR channels. While NPPB blocked *I*_sc_ almost completely, the subsequent addition of amiloride showed an additional but minor reduction on *I*_sc_. (FSK + CFTR_inh_‐172 21.53 ± 2.0 *μ*A/cm^2^, FSK + CFTR_inh_‐172/NPPB 5.21 ± 1.8 *μ*A/cm^2^, and FSK + CFTR_inh_‐172/NPPB/amiloride 3.94 ± 1.9 *μ*A/cm^2^, mean ± SEM, *N* = 6, paired Student's *t*‐test *P* = 0.0021 FSK vs. FSK/CFTR_inh_‐172, *P* < 0.0001 FSK/CFTR_inh_‐172 vs. FSK/CFTR_inh_‐172/NPPB, and *P* = 0.0023 FSK/CFTR_inh_‐172/NPPB vs. FSK/CFTR_inh‐_172/NPPB/amil).

### Effect of NPPB and amiloride on *I*_sc_ at asymmetrical Cl^−^ concentrations

**Figure 5.**
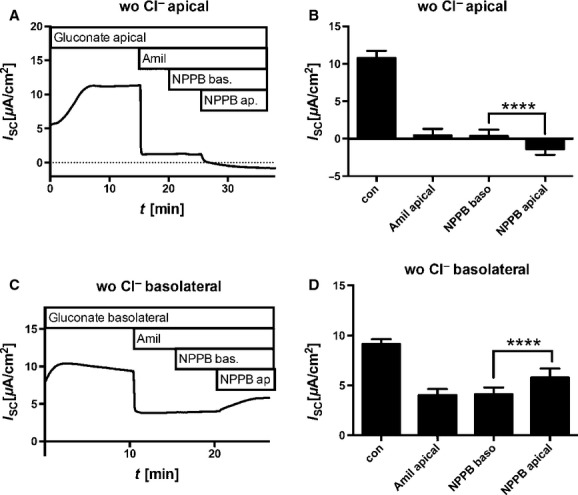
Effect of Cl^−^ concentration gradients on *I*_SC_. Cells were cultivated in the presence of 30 nmol/L dexamethasone at air–liquid interface, mounted into Ussing chambers, and *I*_SC_ measurements were performed. Cl^−^ was replaced by gluconate either on the apical side (A and B, wo Cl^−^ apical) or on the basolateral side (C and D, wo Cl^−^ basolateral). Representative *I*_SC_ measurements are shown in (A) and (C). Time intervals at which compounds were added to the basolateral (bas.) or apical (ap.) solution are given above each curve. Bar diagrams (B and D) summarize measured *I*_SC_ as mean ± SEM. NPPB acts only from the apical side. Its effect on *I*_SC_ depends on orientation of Cl^−^ concentration gradient. Amil = 30 *μ*mol/L amiloride added to the apical side, NPPB bas. = 200 *μ*mol/L 5‐nitro‐2‐(3‐phenylpropylamino)benzoic acid added to the basolateral side, and NPPB ap. = 200 *μ*mol/L 5‐nitro‐2‐(3‐phenylpropylamino)benzoic acid added to the apical side.

The present data are indicative that the effect of NPPB is not additive to the amiloride‐induced inhibition of *I*_sc_ and vice versa. In order to confirm that both inhibitors affect different targets, their effect in the presence of asymmetric Cl^−^ concentrations was investigated (Fig. 5). In these experiments, the driving forces of transepithelial Cl^−^ currents were altered by substituting Cl^−^ with gluconate either in the apical or in the basolateral compartment; the Na^+^ concentration remained unaffected, and thus symmetrical. Amiloride reduced *I*_sc_ regardless of the Cl^−^ concentration gradient. NPPB was added in the presence of amiloride, and affected *I*_sc_ only when applied to the apical side. Its effect on *I*_sc_ was dependent on the Cl^−^ gradient. When Cl^−^ was depleted from the apical compartment, NPPB further reduced *I*_sc_ (Fig. 5A and B, paired Student's *t*‐test DIOA basolateral vs. DIOA apical *P* < 0.00001). When Cl^−^ was depleted from the basolateral compartment, NPPB increased the *I*_sc_ (Fig. 5C and D, paired Student's *t*‐test DIOA basolateral vs. DIOA apical *P* < 0.00001).

### Effect of DIOA on *I*_sc_

**Figure 6.**
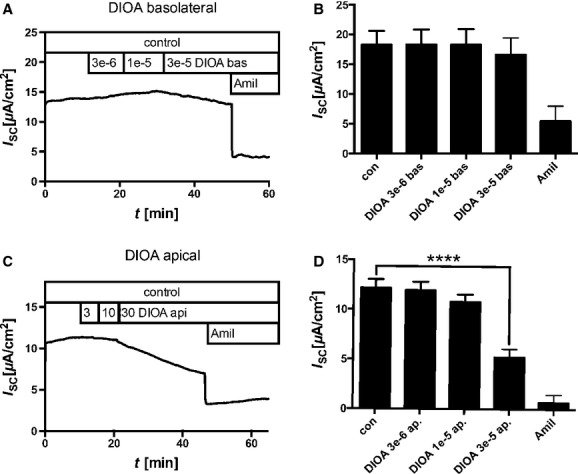
Effect of DIOA on *I*_SC_. Cells were cultivated in the presence of 30 nmol/L dexamethasone at air–liquid interface, mounted into Ussing chambers, and *I*_SC_ measurements were performed. (A) Time course of representative *I*_SC_ measurement. DIOA was added to the basolateral compartment. Numbers give concentrations in mol/L. Amil = 30 *μ*mol/L amiloride added to the apical compartment. (B) Bar diagram summarizes measurements as mean ± SEM (DIOA added to the basolateral and amiloride to the apical compartment). (C) Time course of representative *I*_SC_ measurement. DIOA was added to the apical compartment. Numbers represnt concentrations in mol/L. Amil = 30 *μ*mol/L amiloride added to the apical compartment. (D) Bar diagram summarizes measurements as mean ± SEM (DIOA and amiloride were added to the apical compartment).

Transcellular Cl^−^ transport in lung epithelia was previously hypothesized to depend on basolaterally localized K^+^/Cl^−^ transporters of the SLC12A subfamily. DIOA, a blocker of K^+^/Cl^−^ transporters, did not inhibit *I*_sc_ when applied to the basolateral side (Fig. 6A and B). However, when applied to the apical side, it significantly inhibited *I*_sc_ at a concentration of 30 *μ*mol/L (Fig. 6C and D, paired Student's *t*‐test control vs. 10^−5^ mol/L DIOA *P* < 0.0001). Thus, transcellular ion transport does not depend on basolaterally localized DIOA‐sensitive K^+^/Cl^−^ cotransporters.

## Discussion

Although transepithelial water transport is one of the most important functions in respiratory epithelia, its systematical investigation is still limited. This is not only due to methodological restrictions but also due to the delicate structure of the lung itself. Recent studies on alveolar water clearance were performed either on whole lung (Fang et al. 2002; Mutlu et al. 2004, 2005) or on isolated primary alveolar cells (Fang et al. 2006; Li et al. 2012). These studies revealed that transcellular Na^+^ resorption work in conjunction with transcellular Cl^−^ resorption. Therein, CFTR channels were highlighted as modulators of ENaC‐dependent Na^+^ resorption. Our study aims on the question that to which extent Cl^−^ channels affect ENaC‐dependent transepithelial water resorption.

The herein investigated epithelia are generated from the small cell lung cancer cell line NCI‐H441 (O'Reilly et al. 1989; Wispé et al. 1990), which was introduced as a cellular model of the alveolar epithelium (Hermanns et al. 2004) and expresses major transport pathways of the alveolar epithelium (O'Reilly et al. 1989; Ito et al. 2001; Lee et al. 2003). Even though cell lines should be generally considered with caution as models for native epithelia, their major advantage is to generate homogenous epithelial cell layers with high reproducibility that reflect major epithelial transport pathways.

We recently reported a D_2_O dilution method, which enables the quantification of water transport across cell layers with yet unprecedented volume resolution (Neubauer et al. 2013). This method utilizes the fact that D_2_O and H_2_O can be reliably distinguished using midrange IR spectroscopy. H_2_O and/or D_2_O content of their mixtures can be quantified by analyzing absorption bands of their bending and stretching vibrations. Beside its precision, the major advantages of this method are as follows: (1) it allows to measure the solvent (water) of apical volumes directly and hence it works independent of any markers usually used to estimate changes in aqueous volume due to water transport‐dependent changes in concentration. (2) It exclusively determines vectorial water flux, which depends on the net direction of active ion transport. This is fundamentally different from electrical measurements (transepithelial voltage, *I*_sc_), where Na^+^ resorption cannot – per se – be distinguished from Cl^−^ secretion.

Dexamethasone increases ENaC expression, ENaC‐mediated ion, and water transport (Neubauer et al. 2013). We demonstrated that ion transport increases with dexamethasone concentration, whereas ENaC‐driven water transport reaches saturation at dexamethasone concentrations exceeding 100 nmol/L (Neubauer et al. 2013). Therefore, H441 epithelia were cultivated in the presence of 30 nmol/L dexamethasone. At these conditions, the water transport is not saturated, and is affected by even smaller changes in ion transport.

Our major finding is that ENaC‐mediated water resorption is limited by apically localized Cl^−^ channel activity. A transport‐limiting effect of Cl^−^ channels on water resorption was discussed previously (Fang et al. 2002; Mutlu et al. 2005). The strongest evidence for such a mechanism rose from water transport studies in mice lung in which NPPB reduced water resorption to similar extend as amiloride (Fang et al. 2002), which is in line with our observations. When NPPB was added subsequently to amiloried, it hardly reduced *I*_sc_. The same was observed for amiloride, when subsequently added after NPPB. These observations do not rule out an additive transcellular Cl^−^ transport to ENaC mediated transcellular Na^+^ transport. However, an interdependence of ENaC‐mediated transport on Cl^−^ channel activity is much more evident.

Furthermore, we demonstrate that CFTR work in conjunction with non‐CFTR channels on water resorption and ion transport. Whereas non‐CFTR channels have a rate‐limiting function, CFTR channels act rather as modulators on water transport. Such a modulatory function of CFTR channels on water resorption was already reported (O'Grady et al. 2000; Mutlu et al. 2004, 2005; Fang et al. 2006). Remarkably, blockage of CFTR channels were reported to have no effect on basal water transport (Fang et al. 2006). Due to the unprecedented volume resolution achieved with the D_2_O dilution method, it was possible to demonstrate that CFTR_inh_‐172 and glybenclamide reduced even basal water resorption to a minor, yet significant extent. Herein, it is now shown that CFTR contributes even to basal water resorption. This is in line with measurements on intact small airways, which demonstrated that CFTR channels are constitutively active and contribute to electrogenic transport in these epithelia (Wang et al. 2005).

The observed FSK‐induced increase in water and Na^+^ resorption observed in our study may be explained by modulating ENaC directly. Several lines of evidence support such a mechanism. (1) FSK itself facilitates ENaC incorporation into the apical membrane of cortical collecting duct cells, and therefore, it increases amiloride‐sensitive *I*_sc_ (Edinger et al. 2012; Robins et al. 2013). (2) Terbutaline increases the opening probability of ENaC via cAMP, and thus, increases alveolar fluid clearance (Downs et al. 2012). (3) FSK increases ENaC‐dependent *I*_sc_ in H441 epithelia, which was proposed to depend on increased ENaC incorporation into the apical plasma membrane (Woollhead and Baines 2006).

However, direct activation of ENaC by FSK would not depend on Cl^−^ channels, and hence, FSK‐induced increase in water resorption should be insensitive to Cl^−^ channel modulators like CFTR_inh_‐172 or glybenclamide. Both inhibitors reduced water resorption in FSK‐stimulated cells but only to basal levels observed in nonstimulated control cells. A similar effect was observed for CFTR_inh_‐172 on FSK‐induced *I*_sc_. Although the effects of FSK and CFTR_inh_‐172 on water resorption and *I*_sc_ were similar, they differed significantly in their quantity. The *I*_sc_ measurement quantifies ion transport and the effect of ion channel modulators directly. Ion channels mediate active transport processes, which drive water resorption and hence ion channel modulators acts on water transport rather indirectly. Furthermore, there is not a linear correlation between water transport and electrogenic ion transport (Neubauer et al. 2013). These observations do not disprove a direct FSK activation of ENaC. However, the remaining water resorption in the presence of NPPB is almost the same in FSK‐stimulated and ‐unstimulated control cells. This gives evidence that FSK activation of water resorption acts via Cl^−^ channels, most likely via CFTR rather than via direct ENaC activation.

Consistent with our observation of apically localized Cl^−^ channels, it was demonstrated that Cl^−^ uptake can be modulated by apically localized CFTR channels in distal respiratory epithelia (Kim et al. 1991; Lee et al. 2003). Cl^−^ release into the basolateral compartment was hypothesized to be mediated by K^+^/Cl^−^ cotransporters of the SLC12A family (KCC transporters) (Lee et al. 2003). We observed that DIOA, which was recently introduced as a KCC blocker (Garay et al. 1988), inhibited water transport and *I*_sc_. However, contrary to expectations (Lee et al. 2003), we found no effect in blocking *I*_sc_ in Ussing chamber experiments when DIOA was applied to the basolateral compartment, whereas it inhibited *I*_sc_ significantly when applied to the apical side. Therefore, it is unlikely that basolateral KCC transporters are involved directly in transcellular ion transport. Instead, these observations might suggest an involvement of apically localized KCC transporters, as DIOA blocks KCC transporters with an IC_50_ of 10 *μ*mol/L (Garay et al. 1988). However, several types of Cl^−^ channels may also be blocked by DIOA with a similar potency (Ito et al. 2001; Bräuer et al. 2003). With respect to our observation that NPPB affects *I*_sc_ only when it is applied to the apical side DIOA possibly acts via apically localized Cl^−^ channels.

By use of a novel and powerful technique to quantify transepithelial water transport and by correlating vectorial water transport with electrogenic ion transport, this study revealed a tight interdependency between ENaC‐mediated transports and apically localized Cl^−^ channels. Both channel activities are rate limiting for water resorption in H441 cells, which form a resorptive epithelium. The implication of this finding is that resorptive solute transport, for both Na^+^ and Cl^−^ ions, predominates through the transcellular over the paracellular pathway. The observed interdependency between Cl^−^ channel activity and ENaC herein is shared by primary cultivated alveolar epithelia cells (O'Grady et al. 2000; Jiang and Ingbar 2001; Fang et al. 2006) and has also been observed in whole‐lung experiments (Fang et al. 2002; Mutlu et al. 2005). Even though it was already recognized that NPPB inhibits fluid absorption in whole‐lung experiments to a similar extent as amiloride (Fang et al. 2002), investigators focused on the role of CFTR channels rather than on other Cl^−^ channels. Here, we linked the NPPB effect on water resorption to the blockage of CFTR and non‐CFTR Cl^−^ channels and suggest that the interdependency between Cl^−^ channels and ENaC‐mediated transport is the result of a high cellular Cl^−^ conductance, redirecting Cl^−^ flow from the paracellular shunt through the cells. Consequently, this study extends Cl^−^ channel function in respiratory epithelia from merely a modulatory role toward a rate‐limiting function on water resorption, at least in NCI‐H441 epithelia. The molecular nature of these Cl^−^ channels remains to be determined.

## Conflict of Interest

None declared.
